# Risk Factors and Outcome of Acute Kidney Injury following Acute Myocardial Infarction—A Case Series Study from 2009 to 2019

**DOI:** 10.3390/jcm11206083

**Published:** 2022-10-15

**Authors:** Wen-Hwa Wang, Yu-Cyuan Hong, Hsiu-Min Chen, David Chen, Kai-Che Wei, Ping-Chin Lai

**Affiliations:** 1 Department of Cardiology, Kaohsiung Veterans General Hospital, Kaohsiung 813, Taiwan; 2 Health Management Center, Kaohsiung Veterans General Hospital, Kaohsiung 813, Taiwan; 3 Institute of Management, I-Shou University, Kaohsiung 840, Taiwan; 4 The Kidney Institute and Division of Nephrology, China Medical University Hospital, Taichung 404, Taiwan; 5 Department of Medical Education, Research Center of Medical Informatics, Kaohsiung Veterans General Hospital, Kaohsiung 813, Taiwan; 6 Department of Biomedical Science, Southern Illinois University, Carbondale, IL 62901, USA; 7 School of Medicine, College of Medicine, National Yang Ming Chiao Tung University, Taipei 112, Taiwan; 8 Department of Dermatology, Kaohsiung Veterans General Hospital, Kaohsiung 813, Taiwan

**Keywords:** acute kidney injury, acute myocardial infarction, hemodialysis

## Abstract

**Background:** Historically, acute kidney injury (AKI) has been a common severe complication of acute myocardial infarction (MI). As percutaneous coronary interventions have become more widely used, AMI outcomes have significantly improved. However, post-AMI AKI epidemiology and its associated factors are not well-understood in the age of interventional cardiology. **Materials and methods:** This is a retrospective study examining changes in creatinine levels in all patients admitted for AMI in a single medical center between August 2009 and February 2019. KDIGO criteria were used to define the different stages of post-AMI AKI. **Results:** The study included 1299 eligible cases, among which 213 (16.4%) developed AKI during AMI index admission; and 128 (60.1%), 46 (21.6%), and 39 (18.3%) were classified as KDIGO stages 1, 2, and 3, respectively. Compared with non-AKI subjects, the AKI group had a higher prevalence of non-STEMI (48.4% vs. 29.1%, *p* < 0.001), higher Killip class (3 or 4), and higher in-hospital mortality (15% vs. 2.5%, *p* < 0.001). During the index MI hospitalization, 13.6% (29/213) of the post-MI AKI patients received hemodialysis. Baseline abnormal creatinine (≥1.5 mg/dL), dyslipidemia, and more advanced KDIGO stages (2 or 3) were associated with an increased risk of requiring in-hospital hemodialysis. Moreover, a more advanced KDIGO stage (≥2) was correlated with higher all-cause in-hospital mortality. **Conclusion:** AMI patients remain at risk of AKI, which negatively affects their survival in the modern age.

## 1. Introduction

Acute kidney injury (AKI) can occur following an acute myocardial infarction (AMI), a serious and potentially fatal outcome [1,2,3]. AKI increases short- and long-term mortality in patients with AMI. There is also a possibility of developing post-AMI AKI, regardless of whether the initial renal function is normal or not. Furthermore, a wide variation in the incidence of AKI following AMI, ranging from 5.2% to 59%, has been observed [4].

Due to the widespread use of interventional cardiology in recent decades, the mortality rate of AMI has significantly declined [5]. However, its epidemiology and risk factors have not been well-assessed in the modern era of interventional cardiology. Moreover, the contrast medium used during fluoroscopic coronary angioplasty may damage the kidneys and increase the risk of AKI. For example, one study found that the rate of AKI increased by 20% once patients received primary PCI [6]. As a result, it is crucial to identify patients at risk of developing AKI, as these findings will directly influence physicians’ treatment plans for AMI and, as a consequence, patient outcomes.

Therefore, we conducted this study to clarify the epidemiology and risk factors of AKI following AMI in a single medical center in southern Taiwan between 2009 and 2019. 

## 2. Methods

This study followed the guidelines of the Declaration of Helsinki. The Ethics Committee and the Institutional Review Board of Kaohsiung Veterans General Hospital approved this study (IRB approval number: 20-CT3-10 (200206-1)) and waived the informed consent for study participation. This retrospective cohort study included emergency-room AMI patients (age ≥ 20 years old) who were admitted in Kaohsiung Veterans General Hospital between August 2009 and February 2019.

Basic demographic data, including comorbidities (hypertension, diabetes mellitus, and dyslipidemia), were collected. We excluded cases whose creatinine tests were not performed in our hospital within the past 3 months of the AMI index date. In general practice for AMI patients, creatinine levels were obtained on the first day of admission and were measured every 2–3 days during hospitalization. The baseline and following creatinine levels were analyzed. We used KDIGO (Kidney Disease: Improving Global Outcomes) criteria to define the various stages of AKI, which is defined as increment in serum creatinine by ≥0.3 mg/dL within 48 h; or increment in serum creatinine ≥1.5 times baseline, which is known or presumed to have occurred within the prior 7 days; or urine volume < 0.5 mL/kg/h for 6 h. The staging of AKI is defined as follows: AKI stage 1 as serum creatinine 1.5–1.9 times baseline or ≥0.3 mg/dL increment or urine output less than 0.5 mL/kg/h for 6–12 h; AKI stage 2 as serum creatinine 2.0–2.9 times baseline or urine output less than 0.5 mL/kg/h for more than 12 h; and stage 3 as serum creatinine more than 3.0 times baseline or increment in serum creatinine to ≥4.0 mg/dL or initiation of renal replacement therapy or in patients <18 years, decrease in eGFR to <35 mL/min per 1.73 m^2^ or urine output less than 0.3 mL/kg/h for ≥24 h, or anuria of more than 12 h.

We displayed all data as the mean (±standard deviation) for continuous variables and as the number (percentage) of patients according to different categorical variables. AKI survival-associated factors after MI were analyzed by a bidirectional stepwise model of Cox regression, whereas a Kaplan–Meier survival curve was used to present hemodialysis-free and overall survival in the hospital. SPSS ver. 18 (SPSS Inc., Chicago, IL, USA) was used for statistical analysis. A *p*-value < 0.05 was regarded as a significant level.

## 3. Results

**Figure 1:**
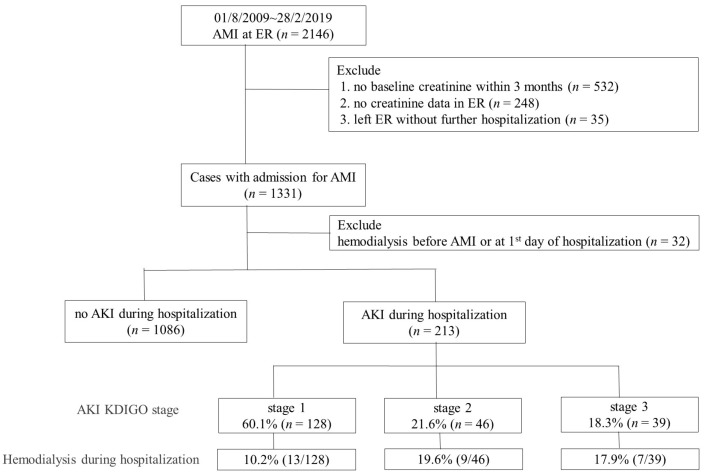
Algorithm and summarized results of the study. AMI: acute myocardial infarction; AKI: acute kidney injury; Cr: creatinine; ER: emergency room. AKI stage 1 as serum creatinine 1.5–1.9 times baseline or ≥0.3 mg/dL increment or urine output <0.5 mL/kg/h for 6–12 h; stage 2 as serum creatinine 2.0–2.9 times baseline or urine output <0.5 mL/kg/h for more than 12 h; stage 3 as serum creatinine more than 3.0 times baseline or increment in serum creatinine to ≥4.0 mg/dL or initiation of renal replacement therapy or urine output less than 0.3 mL/kg/h for ≥24 h, or anuria more than 12 h.

A total of 1299 eligible patients were included in the analysis (Figure 1). A total of 213 patients (213/1299, 16.4%) had AKI during the index AMI hospitalization; whereas 128 (60.1%), 46 (21.6%), and 39 (18.3%) were classified as KDIGO stages 1, 2, and 3, respectively (Table 1). Detailed distributions of baseline and change in creatinine levels for all subjects are shown in Supplementary Figure S1.

AMI patients with AKI had an elevated baseline creatinine level (2.21 ± 1.70 vs. 1.35 ± 1.00 mg/dL), had a greater prevalence of diabetes mellitus (56.8% vs. 33.7%), had a higher baseline renal impairment (54.5% vs. 19.2%), and were older (71.7 ± 12.6 vs. 64.0 ± 14.0) than those without AKI (all *p* < 0.001) (Table 1). When MI-directed factors were analyzed, a higher incidence of non-STEMI (48.4% vs. 29.1%, *p* < 0.001) and a Killip class of 3–4 (70.9% vs. 28.8%, *p* < 0.001) were noted in patients with AKI. Furthermore, similar percentages of patients with and without AKI received PCI or coronary artery bypass grafting (CABG) during their index AMI admissions. In addition, the in-hospital mortality of AKI patients was significantly higher than in non-AKI patients (15% vs. 2.5%; *p* < 0.001) (Table 1).

Among the AKI group, 29 cases (13.6%) received hemodialysis during the index AMI admission. Analysis of the associations between patients’ demography and AKI revealed that an abnormal baseline creatinine (≥1.5 mg/dL), dyslipidemia, and a higher AKI stage (≥2) were associated with a greater need for in-hospital hemodialysis (Table 2).

**Figure 2:**
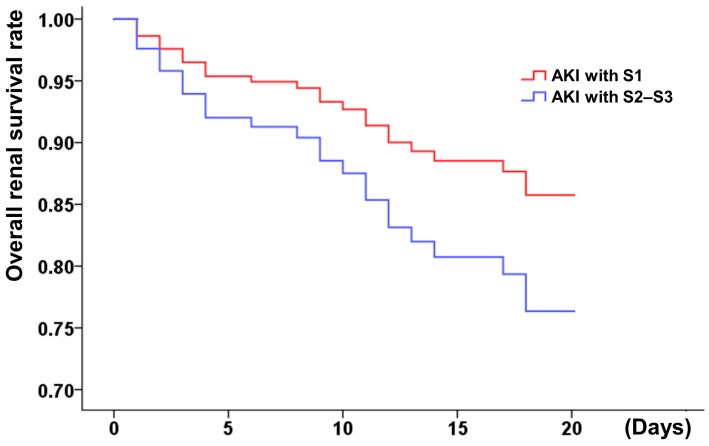
Hemodialysis-free survival of MI patients with different AKI stages (S1: stage 1; S2–S3: stage 2 or 3). AKI stage 1 as serum creatinine 1.5–1.9 times baseline or ≥0.3 mg/dL increment or urine output <0.5 mL/kg/h for 6–12 h; stage 2 as serum creatinine 2.0–2.9 times baseline or urine output <0.5 mL/kg/h for more than 12 h; stage 3 as serum creatinine more than 3.0 times baseline or increment in serum creatinine to ≥4.0 mg/dL or initiation of renal replacement therapy or urine output less than 0.3 mL/kg/h for ≥24 h, or anuria more than 12 h.

**Figure 3:**
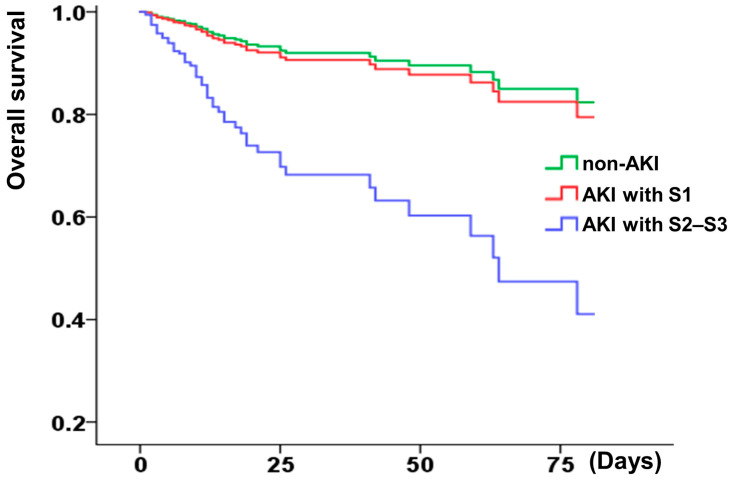
Kaplan–Meier curves display overall survival rate of patients with or without AKI (S1: stage 1; S2–S3: stage 2 or 3). AKI stage 1 as serum creatinine 1.5–1.9 times baseline or ≥0.3 mg/dL increment or urine output <0.5 mL/kg/h for 6–12 h; stage 2 as serum creatinine 2.0–2.9 times baseline or urine output <0.5 mL/kg/h for more than 12 h; stage 3 as serum creatinine more than 3.0 times baseline or increment in serum creatinine to ≥4.0 mg/dL or initiation of renal replacement therapy or urine output less than 0.3 mL/kg/h for ≥24 h, or anuria more than 12 h.

Afterward, the hemodialysis-free survival and the overall short-term survival were analyzed. Higher AKI stages (≥2) correlated with worse overall and renal survival (Figure 2 and Figure 3), whereas KDIGO stage 1 was not associated with decreased overall survival. A correlation analysis was conducted using the demographic characteristics of the patients (described in Supplementary Table S1). The risk of in-hospital hemodialysis was greater in patients with a baseline creatinine of 1.5 mg/dL, dyslipidemia, and a higher stage of AKI (Table 2). In addition to Killip stage (3 or 4) (adjusted HR: 2.61, 1.27–5.36), AKI stage (≥2 or 3) was associated with a higher risk of in-hospital all-cause mortality (HR: 3.58, 1.97–6.49) (Table 3).

## 4. Discussion

Our study indicates that AKI remains a serious complication of AMI in the modern era and negatively impacts survival. Dyslipidemia, KDIGO ≥ 2, and an abnormal creatinine level (≥1.5 mg/dL) at baseline are associated with a poorer hemodialysis-free survival rate. Moreover, Killip stage (3 or 4) and KDIGO ≥ 2 were associated with higher all-cause mortality rates in hospitals. 

The mechanisms of AKI following AMI are complex and multifactorial. AMI can result in myocardial stunning and myocardial hibernation, resulting in unstable hemodynamics, thereby reducing effective blood flow to kidneys, which stimulates the renin-angiotensin system. Inflammatory factors and oxygen free radicals are consequently increased, resulting in AKI [7]. AKI may also be caused by toxins or chemokines released by damaged cardiomyocytes following AMI [8]. Furthermore, the application of the contrast medium might exacerbate AKI through several different mechanisms, such as preexisting dehydration, reducing scavenge nitric oxide and impairing endothelial cell function [9].

Through discrete mechanisms, nephrotoxic agents such as the contrast medium, PPIs, etc. could induce AKI during acute coronary syndromes. Among them, the contrast medium is commonly used in PCIs, which becomes popular in modern cardiology practice. The incidence of AKI after intra-arterial contrast medium injection was 4.0–28.9%, which is highly variable among different patient groups such as the general population and patients with diabetes or chronic kidney disease [10,11]. In a study focus on STEMI patients, Silvain et al. demonstrated that one-year patient mortality was independently predicted by hemodynamic instability, cardiac arrest, preexisting renal failure, elderly age, and a high contrast medium volume. Notably, they also found that the volume of the contrast medium used was not correlated with the elevation of serum creatinine or decrease in the estimated glomerular filtration rate [12]. Due to this complexity, universal measures to prevent postcontrast medium AKI are still lacking. Further pieces of evidence are needed to explore the impact of the contrast medium in AKI post-AMI.

In our study, non-STEMI patients had a higher risk of developing AKI than STEMI patients. A greater proportion (63.3%) of those undergoing hemodialysis during hospitalization were diagnosed with non-STEMI. Although there is no clear explanation for the higher prevalence of non-STEMI in AKI patients, we suspect that early coronary reperfusion intervention, such as primary PCI, plays a significant role. The benefits of primary PCI have been demonstrated in STEMI patients, and it has become the standard of care for STEMI patients. However, for non-STEMI patients, primary PCI is not routinely performed. As early coronary reperfusion benefits myocardial cells, it is reasonable to assume that primary PCI may have a similar effect on renal cells. Further studies should be conducted to determine whether early reperfusion interventions reduce the risk of AKI in non-STEMI patients.

We discovered that 2.31% of MI patients had undergone hemodialysis during their hospitalization for AMI. Furthermore, patients with more advanced stages of AKI would receive more frequent treatments of hemodialysis. Additionally, baseline abnormal creatinine (≥1.5 mg/dL) was significantly associated with renal failure, which requires hemodialysis. It is worth noting that some of these patients initially had normal creatinine levels but developed AKI later (Supplementary Figure S1). Therefore, cardiologists should monitor renal function more frequently during AMI hospitalization, even if the patient initially appears to have a normal creatinine level.

## 5. Conclusions

The risk of AKI still remains among AMI patients in the era of intervention cardiology, despite the fact that the overall mortality and complications associated with AMI have decreased over the years. Therefore, identification of the risk factors for AKI following AMI is imperative. Physicians should be more attentive to the drug dosage adjustment according to the AKI stage, avoid prescribing nephrotoxic medications, such as NSAIDs, and reduce the contrast medium volume used during PCIs.

## 6. Limitation

First, the study lacked long-term follow-up of renal function, as some patients may not have undergone follow-up after discharge. Second, this study graded AKI using the KDIGO criteria despite other definition systems being available. In the past, AKI was simply defined as an increase in serum creatinine concentration of more than 25% or 0.5 mg/dL within 48–72 h [13]. Various rigorous definitions were developed later, such as “the risk, injury, failure, loss, and end-stage kidney disease (RIFLE) criteria”, which were later modified to AKIN criteria [14,15]. In this study, KDIGO criteria were used because they were reported to be more sensitive in investigating high-risk patients, including AMI patients [16,17,18,19]. Some of our patients in this study stayed in the hospital for less than 7 days; therefore, the incidence of AKI may be underestimated in this group of patients. The impact of hemodynamic changes such as arterial pressure, cardiac output, and cardiac intervention on renal survival was not analyzed.

## Figures and Tables

**Table 1:** Demographics and clinical characteristics of patients in non-AKI and AKI groups.

Variables	Non-AKI *n* = 1086 (%)	AKI *n* = 213 (%)	*p*-Value	H/D *n* = 30 (%)
Age (y/o, mean ± SD)	64.0 ± 14.0	71.7 ± 12.6	<0.001	70.5 ± 13.4
≥65 y/o	514 (47.3)	151 (70.9)	<0.001	19 (63.3)
Male gender	898 (82.7)	157 (73.7)	0.002	22 (73.3)
Baseline Cr level (mg/dL)	1.35 ± 1.00	2.21 ± 1.70	<0.001	3.75 ± 2.04
Baseline renal impairment ^§^	209 (19.2)	116 (54.5)	<0.001	27 (90.0)
Comorbidities				
Hypertension	613 (56.4)	121 (56.8)	0.922	20 (66.7)
Diabetes mellitus	366 (33.7)	121 (56.8)	<0.001	21 (70.0)
Dyslipidemia	416 (38.3)	62 (29.1)	0.011	17 (56.7)
AMI types				
STEMI	610 (56.2)	80 (37.6)	<0.001	9 (30.0)
Non-STEMI	316 (29.1)	103 (48.4)		19 (63.3)
Unclassified	160 (14.7)	30 (14.1)		2 (6.7)
AMI severity/treatment				
Killip 3–4	313 (28.8)	151 (70.9)	<0.001	25 (83.3)
Heart resuscitation intervention ^¶^	645 (59.4)	116 (54.5)	0.181	9 (30.0)
Outcome				
Hemodialysis during hospital	-	29 (13.6)	-	-
Mortality in hospital	27 (2.5)	32 (15.0)	<0.001	-
One year mortality ^¥^	20 (1.9)	8 (4.4)	0.034	-

Cr: creatinine; STEMI: ST-elevation myocardial infarction; H/D: hemodialysis; SD: standard deviation. ^§^ Baseline creatinine was ≥1.5 mg/dL before the attack of AMI. ^¥^ Subjects with mortality in hospital (*n* = 59) had been excluded for statistics. ^¶^ Including primary percutaneous coronary intervention (PCI) and coronary artery bypass graft (CABG).

**Table 2:** Stepwise Cox regression analysis for in-hospital hemodialysis in AMI patients.

Variables	Adjusted HR	95% CI	*p* Value
Baseline creatinine ≥ 1.5 mg/dL	10.27	3.06–34.49	<0.001
Dyslipidemia	2.93	1.41–6.09	0.004
AKI stage	4.51	2.73–7.46	<0.001

HR: hazard ratio; CI: confidence interval.

**Table 3:** Bidirectional stepwise Cox regression analysis for all-cause in-hospital mortality in MI patients.

Variables	Adjusted HR	95% CI	*p* Value
Killip stage 3–4	2.61	1.27–5.36	0.009
AKI stage			
None	1.00	-	-
1	1.01	0.45–2.27	0.980
2–3	3.58	1.97–6.49	<0.001

HR: hazard ratio; CI: confidence interval. AKI stage 1 as serum creatinine 1.5–1.9 times baseline or ≥0.3 mg/dL increment or urine output <0.5 mL/kg/h for 6–12 h; stage 2 as serum creatinine 2.0–2.9 times baseline or urine output <0.5 mL/kg/h for more than 12 h; stage 3 as serum creatinine more than 3.0 times baseline or increment in serum creatinine to ≥4.0 mg/dL or initiation of renal replacement therapy or urine output less than 0.3 mL/kg/h for ≥24 h, or anuria more than 12 h.

## Supplementary Materials

The following supporting information can be downloaded at: https://www.mdpi.com/article/10.3390/jcm11206083/s1, Figure S1: Detailed distributions of baseline and change in creatinine levels for all subjects; Table S1: Demographics and clinical characteristics of patients with mortality in hospital (*n* = 1299).
