# Supplementary material for: Risk Factors and Outcome of Acute Kidney Injury following Acute Myocardial Infarction—A Case Series Study from 2009 to 2019
Source: J Clin Med. 2022 Oct 15;11(20):6083. doi: 10.3390/jcm11206083 (PMC9604918; doi:10.3390/jcm11206083)
Supplement: Supplementary file 1 [file jcm-11-06083-s001.zip › jcm-1906585-supplementary.pdf]

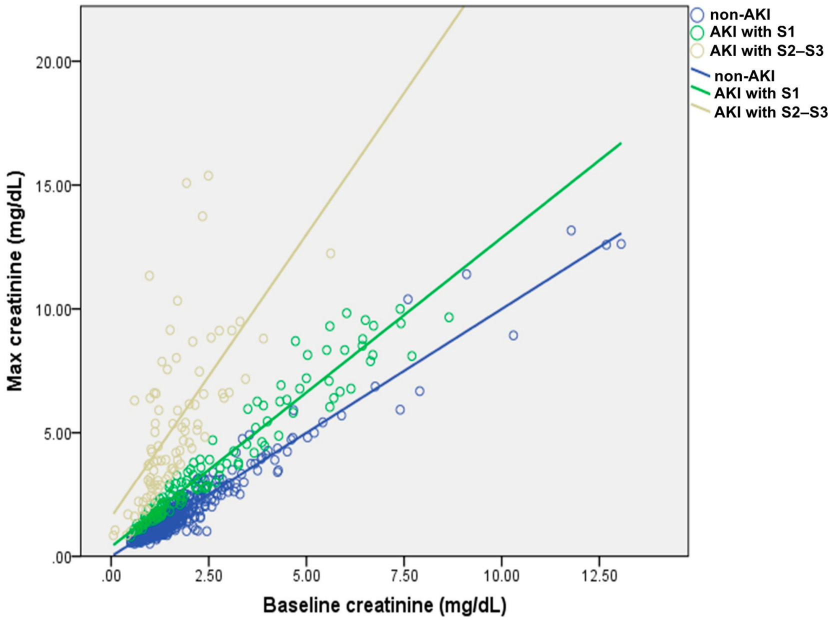

Figure S1. Detailed distributions of baseline and change in creatinine levels for all subjects.

Table S1. Demographics and clinical characteristics of patients with mortality in hospital (n = 1299).

Variables	Survive *n* = 1240 (%)	Dead *n* = 59 (%)	*p*-value
Age	64.9±14.0	73.2±13.7	<0.001
≥ 65 years-old	622 (50.2)	43 (72.9)	0.001
Male, %	1017 (82.0)	38 (64.4)	0.001
Baseline Cr level (mg/dL)	1.48±1.18	1.60±1.30	0.495
Baseline renal impairment §	300 (24.2)	25 (42.4)	0.002
Comorbidities			
Hypertension	698 (56.3)	36 (61.0)	0.474
Diabetes mellitus	459 (37.0)	28 (47.5)	0.106
Dyslipidemia	463 (37.3)	15 (25.4)	0.064
MI directed factors			
Type			0.201
STEMI	665 (53.6)	25 (42.4)	
Non-STEMI	397 (32.0)	22 (37.3)	
missing	178 (14.4)	12 (20.3)	
MI severity			
Killip 3-4, %	416 (33.5)	48 (81.4)	<0.001
Heart resuscitation intervention	728 (58.7)	33 (55.9)	
AKI stage, %	181/1240 (14.6)	32/59 (54.2)	<0.001
None (*n* = 1086)	1059 (97.5)	27 (2.5)	<0.001
1 (*n* = 128)	120 (93.8)	8 (6.2)	
2 (*n* = 46)	32 (69.6)	14 (30.4)	
3 (*n* = 39)	29 (74.4)	10 (25.6)	

STEMI: ST-elevation myocardial infarction, MI: myocardial infarction, SD: standard deviation, PCI: percutaneous coronary intervention, CABG: coronary artery bypass grafting.

§ Creatinine ≥ 1.5 mg/dl.
